# Is a flick-through enough? A content analysis of Advanced Driver Assistance Systems (ADAS) user manuals

**DOI:** 10.1371/journal.pone.0252688

**Published:** 2021-06-17

**Authors:** Oscar Oviedo-Trespalacios, Jennifer Tichon, Oliver Briant

**Affiliations:** 1 Centre for Accident Research & Road Safety–Queensland (CARRS-Q), Queensland University of Technology (QUT), Kelvin Grove, QLD, Australia; 2 Centre for Future Mobility, Queensland University of Technology (QUT), Kelvin Grove, QLD, Australia; Monash University, AUSTRALIA

## Abstract

Advanced Driver Assistance Systems (ADAS) are being developed and installed in increasing numbers. Some of the most popular ADAS include blind spot monitoring and cruise control which are fitted in the majority of new vehicles sold in high-income countries. With more drivers having access to these technologies, it is imperative to develop policy and strategies to guarantee the safe uptake of ADAS. One key issue is that ADAS education has been primarily centred on the user manual which are not widely utilised. Moreover, it is unclear if user manuals are an adequate source of education in terms of content and readability. To address this research gap, a content analysis was used to assess the differences in ADAS-related content and readability among the manuals of the highest selling vehicles in Australia. The qualitative findings showed that there are seven themes in the user manuals: differences between driving with and without ADAS, familiarisation requirements, operational limits of the ADAS, potential ADAS errors, behaviour adaptation warnings, confusion warnings, and malfunction warnings. The quantitative analysis found that some of the manuals require several years of education above the recommended for a universal audience (>8 years) to be understood. Additionally, there is a notable number of text diversions and infographics which could make comprehension of the user manual difficult. This investigation shows that there is a lack of standardisation of ADAS user manuals (in both content and delivery of information) which requires regulatory oversight. Driver ADAS education needs to be prioritised by policymakers and practitioners as smart technology continues to increase across the transport system. It seems that current strategies based on user manuals are insufficient to achieve successful adoption and safe use of these technologies.

## 1. Introduction

A wide range of Advanced Driver Assistance Systems (ADAS) have been developed and installed in vehicles within the past two decades. These technologies are designed to assist drivers with key driving functions including speed management via intelligent speed adaptation systems, and timely braking via autonomous braking systems. Previous studies have found ADAS to increase safety [1, 2]. A recent study by Thompson et al. [3] found the use of collision avoidance technology increased the safety margin during car following interactions through the use of both audio and visual cues. These findings provide support for a vast body of literature focusing on vehicle crash analysis, which also highlights the potential benefits of ADAS [4–6]. With vehicle manufacturers marketing safety as a primary consumer benefit, future vehicles will likely contain ever increasing numbers of these technologies. Therefore, drivers must understand the benefits and have the skills to operate this technology correctly for their own and others’ safety.

The surge of smart vehicle technologies appears to lack any standardisation, as many automakers use this technology to create competitive advantages. As a result, road users now have a variety of options to choose from when selecting a vehicle. While some newer vehicle models contain similar technologies, such as blind-spot monitoring, many of these technologies differ between vehicle brands and models. For example, collision avoidance technology (CAT) varies significantly across vehicle models. CAT systems can use either radar, lidar, a camera, or a combination of these to detect an object or obstacle that the vehicle may collide with [3]. Additionally, the alert for a potential obstruction may differ between makes and models. Some CAT systems automatically apply the brakes to slow the vehicle to avoid a collision. In contrast, other CAT systems alert the driver via an audible or visual notification that the driver must respond to. This lack of standardisation creates inconsistent mental models about the CAT functioning, which then requires time for familiarisation every time a driver is switching to a vehicle with different technology.

Familiarisation with novel ADAS technology is not the only challenge that users face when operating a new vehicle. Labelling irregularities within the vehicle industry introduce confusion into the learning process, whereby users must recognise that different manufacturers often label technology differently. For example, the vehicle’s ability to regulate a consistent speed autonomously while driving may be named ‘Adaptive Cruise Control’ by one company and ‘Distance Assist’ by another. The American Automobile Association [7] examined the number of unique names given by vehicle brands to the same or similar technology. Across 34 brands of vehicles sold in the U.S., there were 20 unique names for adaptive cruise control, 19 for lane-keeping assistance and blind-spot warning, and 18 for automatic high beams, among others [7]. These irregularities could result in confusion, which would undermine the development of accurate mental models about the operation of ADAS.

One key issue is that education and training about ADAS has mainly been the responsibility of the driver. Generally, drivers are required to read the user manuals and complement this with other sources of information, such as internet research. However, reports show that the majority of drivers resort to inaccurate learning methods, rather than utilising user manuals or receiving instructions from the dealer [8]. Indeed, there is an adequate level of consensus in the literature regarding the poor usability and acceptability of user manuals. In an interview study, Viktorová and Šucha [9] found that most drivers do not read the user manual to learn about the technology in their vehicle. Instead, it was found that drivers were relying on a much more dangerous ‘trial and error’ method of learning. Mehlenbacher, Wogalter and Laughery [10] found that 41% of respondents reported having not read the manual for the vehicle they drove most often, despite having access to it. They also reported that, of those that did read it, only 5.2% read upwards of 90% of the manual. Leonard and Karnes [11] similarly found that only 6.8% of their 221 participants claimed to have read their vehicle manual, and 6.3% claimed to have read none of it. The evidence indicating drivers are not reading the manual for vehicles without ADAS sets a worrying precedence for current and future cars, which are equipped with much more sophisticated technology.

A further complication associated with ADAS implementation is that, even if drivers know they possess a system, they often do not understand the functioning or limitations of their ADAS technology. For example, a national cross-sectional study of drivers in Spain found that not knowing how to use the system was one of the main reasons for not using ADAS, including fatigue detection systems and adaptive cruise control [12]. More concerningly, it seems that many drivers can be unaware of the limitations of the ADAS, such as the limitations of cameras, radars and other sensors that are vital to the operation of these technologies, as demonstrated in United States research [13]. It has been suggested that one of the reasons for this is that user manuals are difficult to understand and contain insufficient information [9]. These studies highlight the importance of a user-centred approach for operations manuals, to allow easy comprehension and understanding of the application being used. Therefore, a key gap in the literature is our limited knowledge of the issues presented in user manuals of vehicles fitted with ADAS. An understating of current characteristics in user manuals can provide information on critical issues and opportunities to improve knowledge on the capabilities and risks of ADAS in the community.

The present study aims to analyse a variety of vehicle operations manuals from different brands and to develop a descriptive model of the information presented. User manuals have been used in previous research as effective sources of educational information for operators across a range of industries [10, 14, 15]. This analysis will focus on ADAS technologies, i.e. cruise control and blind-spot monitoring, and will highlight deficits in the delivery of important information about ADAS operation and limitations. The analysis will also examine the comprehensiveness of the manuals, and comment on the shortcomings of technical language for a general target audience.

## 2. Methods

A content analysis was used to assess the differences in ADAS-related content and readability among the manuals of the highest selling vehicles in Australia. This process comprised two sections; the first of which was the search strategy used to locate vehicles that would be eligible for use in the study, and their corresponding user manuals. The second was the data processing used to analyse the content of each user manual.

### 2.1. Search strategy

A search on the Google search engine was performed to identify vehicles equipped with advanced driver assistance systems. The search was designed to identify the most purchased vehicle models in Australia during 2018, as it was surmised that these vehicles would be equipped with more advanced driving systems. The vehicles examined were limited to those listed as popular in Australia, as it has been previously found that vehicles in different countries differ, despite being branded as the same make and model [16]. Therefore, the vehicles examined were limited to those most purchased in Australia.

It was also important that the vehicles used were representative of the vehicles owned by the general population, rather than top-of-the-range models. For example, a recent Tesla model may be fitted with more advanced driver assistance systems than a recent Toyota Hilux, however a brand-new Tesla model would be unaffordable for much of the population. The ten most purchased vehicles in 2018 were considered [17]: Toyota Hilux, Ford Ranger, Mazda 3, Mitsubishi Triton, Mazda CX-5, Toyota Corolla, Hyundai i30, Mitsubishi ASX, Toyota Land Cruiser, Mitsubishi Outlander. The car manuals for each of these vehicles were found on the official page of the vehicle manufacturer. The vehicle manual of the Mitsubishi Triton was not made available on the official Mitsubishi website, nor was any version made available online. As the list of top selling vehicles did not elaborate further than a 10^th^ vehicle with which to replace the missing manual, the Mitsubishi Triton was omitted from the study, and the total number of vehicles examined was nine.

Despite differences between vehicle models, a vehicle owner will receive a manual that covers all models of a vehicle for that year. For example, while a Toyota Hilux TGN1*6 and a Toyota Hilux KUN1*5 will have different engines, the owner will be provided with the same vehicle manual that will include information on both models. and be labelled as the operations manuals for Toyota Hilux. The present study has focused only on these broad, all-inclusive vehicle manuals.

### 2.2. Data processing

The data processing stage comprised a two-part process. The themes were first determined through a deductive approach, wherein the sections pertaining to smart vehicle technology were read by two members of the research team, and relevant sections were noted. These sections were; intelligent speed adaptation/ speed limit warning/adaptive cruise control and blind spot monitoring/warning with lane change assist. From these two sections, broad parent themes were created from the content found across all manuals. The second part of the process utilised an inductive approach, wherein the content of each theme was examined and analysed. Themes that related to driver safety and safe usage of ADAS technologies was retained.

For ease of analysis, Intelligent speed adaptation/Speed limit warning/Adaptive Cruise control and any other advanced driver system that moderates and adapts the speed of the vehicle when in use has been named ‘Cruise Control’. Similarly, any ADAS that monitors the driver’s blind spot or assists the driver in lane changing by checking the surrounding road environment has been named “Blind Spot Monitoring”. Each car manual was assessed on these two applications: cruise control and blind spot monitoring. Within each of these applications, each manual was coded according to seven different themes. The data was coded by three members of the research team. After the qualitative analysis was completed, we conducted the quantitative analysis of the manuals. The results are presented in the order in which the analyses were conducted.

### 2.3. Qualitative data analysis

The content of the manuals was coded according to seven different themes based on the manual content. The themes included:

#### 2.3.1. User manual describes differences between driving with and without ADAS

The theme “*informing differences*” considers when the user manual describes the differences between driving with and without ADAS. This includes when the manual specifies the difference between driving with ADAS in an operational mode or driving with the system disengaged. For example, manually adjusting the vehicle speed rather than using the cruise control technology. Manuals were coded with either ‘Yes’ (present) or ‘No’ (absent). A manual that is coded as ’No’ indicates that it did not specify any difference between driving with or without the technology engaged.

#### 2.3.2. User manual asks users to familiarise with the ADAS information

The theme “*familiarisation*” refers to a notice within the manual itself that advises the owner to become familiar with the manual and the smart technology. This is categorised as any time that any form of advice is given in regard to becoming familiar with any of the specified technology. Manuals will be coded for familiarisation as either ‘Yes’ (present) or ‘No’ (absent). A manual that is coded as ’No’ indicates that no message or warning indicates that the user should become familiar with the instructions for the smart technology and/or the user manual itself.

#### 2.3.3. User manual informs users of the operational limitations of the ADAS

The theme *“operational limits”* considers when the user manual explains the operational limitations of the ADAS, including the warnings messages that indicate when the ADAS should not be used because of its own limitations. This warning could reference the road environment and highlight situations such as rain or snow. Manuals will be coded in regard to application warnings as either ‘Yes’ (present) or ‘No’ (absent). Manuals that are coded as ’Yes’ must have clearly signed warnings that are in bold text and draw the attention of the reader. They must also clearly highlight when the application should and should not be used.

#### 2.3.4. User manual describes potential error modes of the ADAS

The theme *“potential ADAS errors”* refers to the potential situations when the ADAS may present an error when the system is operating. Compared to the operational limits, errors are issues that occur when the ADAS is operating in situation where typically should operate normally. This can take the form of an explanation that highlights an action that may cause an unexpected error and cause the ADAS to cease normal function, or an action that will prevent the application from being activated. Manuals will be coded for potential error warning as either ‘Yes’ (present) or ‘No’ (absent). Manuals that are coded as ’No’ will demonstrate no warnings of potential errors within the system, nor any actions that can cause or remedy a potential error.

#### 2.3.5. User manual informs users of how the ADAS will change their behaviour (behavioural adaptation)

The theme “*behaviour adaptation warnings*” considers warnings regarding behaviour change as a result of using ADAS. This can take the form of a warning that explains how the technology could change their behaviour, and what behaviours the driver will need to retain to maintain safe driving behaviour. Manuals will be coded for behaviour adaptation warnings as either ‘Yes’ (present) or ‘No’ (absent). Manuals that are coded as ’Yes’ display warnings in clearly bolded and noticeable text, and clearly indicate the effect that using the smart technology may have on the driver, and how the driver should behave accordingly.

#### 2.3.6. User manual warns users about confusion when using the system

The theme *“Confusion warnings”* describes a notification within the manual that indicates that there is the possibility that the user may not be aware of a normal function. This can be in the form of a delay, or a sound that the operator may not be aware of, however is part of the normal function of the system. Manuals will be coded for confusion warnings as either ‘Yes’ (present) or ‘No’ (absent). Manuals that are coded as ’No’ indicate that there are no notifications about potential confusion regarding the use of the smart technology within the vehicle.

#### 2.3.7. User manual instructs users how to respond to a malfunction

The theme *“Malfunction warning”* includes instructions of how to respond to a malfunction, e.g. vehicle should be returned to the dealer or manufacturer due to an error. This may also refer to the need to take the vehicle to a repair shop that is licensed to deal with the specific vehicle brand. This theme goes above and beyond the ‘potential errors’ theme, in that these malfunctions are more serious in nature and may endanger the driver. User manuals were coded for malfunction warnings as either ‘Yes’ (present) or ‘No’ (absent). User manuals that are coded as ’No’ indicate that there are no warnings that the vehicle may need to be returned to a dealer or taken to a specialised repairer due to an error or malfunction.

### 2.4. Quantitative data analysis

User manuals were also coded for attributes that could facilitate or make their use and understanding difficult. Three main attributes were identified: diversions, use of infographics, and readability.

#### 2.4.1. Frequency of diversion in the manual

Diversion involves asking users to refer to another section of the manual for further information. Manuals include diversions that can be categorised as ‘outside the section’ and ‘inside the section’. Diversions that are coded as ‘outside the section’ indicate that a message has led the reader to another paragraph or message that is outside the section or chapter that they are currently reading. For example, within the manual for the Mazda CX5, in chapter 4, page 170, there is the message “Refer to Message Indicated on Display on page 7–65” which leads the reader to page 65 of chapter 7 and would therefore be found to be ‘outside the section’. In contrast, on page 172 of chapter 4, the message ‘Refer to Cruise Control Function on page 4–180’ would be found to be ‘within the same section’, as the diversion leads the reader to a part within the same chapter. The measure of section diversion will comprise of the sum total of ‘outside the section’ diversions.

#### 2.4.2. Number of infographics

**Fig 1:**
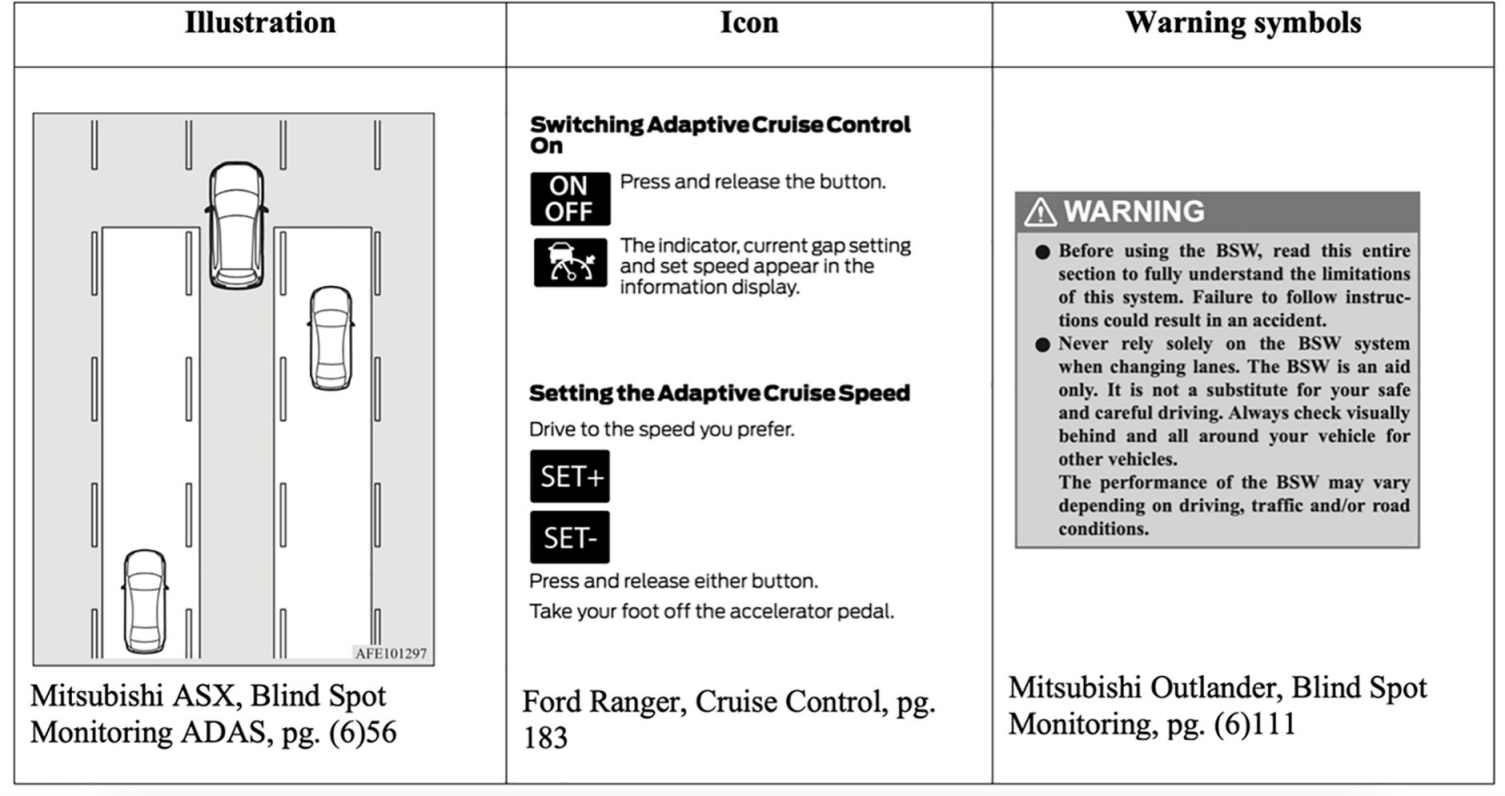
Examples of infographics.

In the context of driving manuals, there are three main types of infographics (see Fig 1): illustrations, icons and warning symbols. An illustration is any diagram or photo that conveys information about the functionality of the vehicle to the reader. An illustration may include text, and contain information related to the vehicle system itself or the road environment. An icon is a small visual representation within the vehicle manual text that conveys the meaning of a button or dashboard light. Icons are used to accurately show the reader what the button or light may mean and will also be an exact replica of the button decal or light shape that the reader will see when operating the vehicle. Warning symbols are small, triangular symbols with an exclamation point in the middle, which will directly proceed the word ‘warning’ or ‘caution’. These are designed to draw the attention of the reader and symbolise that the reader should take note of the text following the warning symbol. Illustrations, icons and warning symbols were all counted independently, and the score of each consists the summed total of each sub-category within the respective ADAS section of the operations manual.

#### 2.4.3. Readability

Readability is the level of ease or difficulty with which text material can be understood by the reader. An online readability calculator was used to determine how easily the manuals can be read by those that purchase the vehicle (https://www.online-utility.org/english/readability_test_and_improve.jsp). The online calculator measures text based on the Gunning Fog index, which indicates the number of years of formal education required to easily read and understand the text on the first read through. Texts intended for a near universal audience, such as vehicle owners, the ideal score is reported to be six to eight. Texts intended for a wide audience generally require a Gunning Fog index of 12 at maximum. Scores above 12 are reported to be too complex for wider audiences to understand with ease. For both cruise control and blind spot monitoring, each section was copied into the readability software in the entirety and the Gunning Fog index was recorded. The entire chapters of cruise control and blind spot monitoring were analysed. Prior to entering the text, each chapter was edited to remove bullet points or any other formatting anomalies that may influence the software’s ability to analyse the text. If there was no formatting, the text was entered into the program without any changes being made. In addition to the overall score, readability analyses were conducted from samples from the beginning, middle and end of the chapters.

## 3. Results

### 3.1. Qualitative analysis

Seven key themes were identified in the manuals. Examples of the quotes found in the user manuals can be seen in Tables 1 and 2. To study the prevalence of these themes, each manual was coded based on a binary ‘present/absent’ coding system, wherein manuals were coded as ‘yes’ if the manual contained information relating to the ADAS theme identified, or ‘no’ if no information was present (see Table 3). There was a great diversity in terms of the information presented within each of the vehicle user manuals. The themes that were found to have the lowest presence rates were the driving without the system and familiarisation themes within the cruise control sections, with each theme being present in only 11.11% of manuals individually. Following this, confusion warnings within the blind spot monitoring sections were found in only 14.29% of the relevant manuals analysed. An important consideration is that Toyota Hilux and Toyota Corolla models were not fitted with blind spot monitoring systems, so they were coded as absent. As a result, the total of vehicle manuals analysed for blind spot monitoring systems, for the purpose of finding means and standard deviations, was considered to be seven rather than nine.

**Table 1:** Qualitative analysis of blind sport monitoring systems.

Theme	Quotes
*Theme 1*. *User manual describes differences between driving with and without ADAS*	“If you switch the blind spot information system off, blind spot information system with trailer tow automatically turns off.” (Ford Ranger, pg. 196)
	“If you turn the BSW ON/OFF, the Rear Cross Traffic Alert (RCTA) also turns ON/OFF at the same time.” (Mitsubishi Outlander, pg. (6)109)
*Theme 2*. *User manual asks users to familiarise with the ADAS information*	“Before using the BSW, read this entire section to fully understand the limitations of this system. Failure to follow instructions could result in an accident.” (Mitsubishi ASX, pg. (6)55; Mitsubishi Outlander, pg. (6)108)
*Theme 3*. *User manual informs users of the operational limitations of the ADAS*	“The BCW range varies relative to vehicle speed. Note that if your vehicle is travelling much faster than the vehicles around you, the warning will not occur.” (Hyundai i30, pg. (5)70)
	“The system does not operate in park (P) or reverse (R).” (Ford Ranger, pg. 195)
	“Under the following conditions, the radar sensors (rear) cannot detect target objects or it may be difficult to detect them.
	A vehicle is in the detection area at the rear in an adjacent driving lane but it does not approach. The BSM determines the condition based on radar detection data.” (Mazda CX5, pg. (4)127)
	“The system may not operate properly during severe weather conditions, for example snow, ice, heavy rain and spray. Always drive with due care and attention. Failure to take care may result in a crash.” (Ford Ranger, pg. 198)
*Theme 4*. *User manual describes potential error modes of the ADAS*	“When the BCW cancelled warning message is displayed in the cluster, check to make sure that the rear bumper is free from any dirt or snow in the areas where the sensor is located. Remove any dirt, snow, or foreign material that could interfere with the radar sensors. After any dirt or debris is removed, the BCW system should operate normally after about 10 minutes of driving the vehicle.” (Hyundai i30, pg. (5)76)
	“The BSM warning indicator light may turn on and the vehicle detection screen may be displayed in the display in reaction to stationary objects (guardrails, tunnels, sidewalls, and parked vehicles) on the road or the roadside.” (Mazda 3, pg. (4)120)
	Due to certain limitations with the operation of this system, the BSM warning indicator light, the warning sound and the warning screen indicator display may not activate or they might be delayed even though a vehicle is in an adjacent driving lane. Always make it your responsibility as a driver to check the rear.” (Mazda CX5, pg. (4)126)
	“In certain situations, the BSW may not detect a vehicle in the detection areas or the detection may be delayed. Some of these include:
	• When a small motorcycle or a bicycle is behind your vehicle.
	• When a vehicle is travelling alongside of your vehicle at nearly the same speed for prolonged periods of time.” (Mitsubishi ASX, pg. (6)56)
*Theme 5*. *User manual informs users of how the ADAS will change their behaviour (behavioural adaptation)*	“**WARNING:** Do not use the blind spot information system as a replacement for using the interior and exterior mirrors or looking over your shoulder before changing lanes. The blind spot information system is not a replacement for careful driving.” (Ford Ranger, pg. 195)
	“The Blind-spot Collision Warning (BCW) system is not a substitute for proper and safe driving. Always drive safely and use caution when changing lanes or backing up the vehicle. The Blind-spot Collision Warning (BCW) system may not detect every object alongside the vehicle.” (Hyundai i30, pg. (5)71)
*Theme 6*. *User manual warns users about confusion when using the system*	“In the following case, the flashing of the BSM warning indicator light, and the activation of the warning sound and the warning screen indicator display may not occur or they may be delayed.
	• A vehicle makes a lane change from a driving lane two lanes over to an adjacent lane. […]” (Mazda 3, pg. (4)119)
	“BCW: Blind spot area. The BCW range varies relative to vehicle speed. Note that if your vehicle is travelling much faster than the vehicles around you, the warning will not occur.” (Hyundai i30, pg. (5)70)
	“If the BSM warning indicator lights turn on when the position lights are turned on, the brightness of the BSM warning indicator lights is dimmed.” (Mazda CX5, pg. (4)130)
*Theme 7*. *User manual instructs users how to respond to a malfunction*	“If the system still does not operate normally have your vehicle inspected by a HYUNDAI authorised repairer.” (Hyundai i30, pg. (5)76)
	“When the warning display appears, the BSW does not operate normally because there are some malfunctions in the system or the sensor. Have the vehicle inspected at a MITSUBISHI MOTORS Authorized Service Point as soon as possible.” (Mitsubishi Outlander, pg. (6)111)

**Table 2:** Qualitative analysis of cruise control systems.

Theme	Quotes
*Theme 1*. *User manual describes differences between driving with and without ADAS*	“**WARNING:** If you override the system by pressing the accelerator pedal, it does not automatically apply the brakes to maintain a gap from any vehicle ahead.” (Ford Ranger, pg. 184)
*Theme 2*. *User manual asks users to familiarise with the ADAS information*	“For your safety, please read the owner’s manual before using the Smart Cruise Control system.” (Hyundai i30, pg. (5)138)
*Theme 3*. *User manual informs users of the operational limitations of the ADAS*	“WARNING: Do not use cruise control on winding roads, in heavy traffic or when the road surface is slippery. This could result in loss of vehicle control, serious injury or death.” (Ford Ranger, pg. 181)
	“Always turn off the cruise control system when it is not in use:
	Leaving the cruise control system in an activation-ready state while the cruise control is not in use is dangerous as the cruise control could unexpectedly activate if the activation button is accidentally pressed, and result in loss of vehicle control and an accident.” (Mazda CX5, pg. (4)237)
	“**WARNING:** Normal cruise control will not brake when your vehicle is approaching slower vehicles. Always be aware of which mode you have selected and apply the brakes when necessary.” (Ford Ranger, pg. 188)
	“Do not use cruise control when driving conditions will not allow you to stay at the same speed, such as in heavy traffic or on roads that are winding, icy, snow-covered, wet, slippery, on a steep downhill slope.” (Mitsubishi Outlander, pg. (6)70)
*Theme 4*. *User manual describes potential error modes of the ADAS*	“Your vehicle may accelerate up to the set speed in the following situations. Apply the brake, if necessary, to slow down.
	• When your vehicle no longer follows the vehicle in front, e.g. at a freeway ex- it or when your vehicle or the vehicle in front changes its lane. (…)” (Mitsubishi Outlander, pg.(6)83)
	“**WARNING:** Adaptive cruise control only warns of vehicles detected by the radar sensor. In some cases there may be no warning or a delayed warning. You should always apply the brakes when necessary. Failure to do so may result in a crash, serious injury or death.” (Ford Ranger, pg. 183)
	“Under certain conditions, the audible alarm may not work at all or may be scarcely audible. Do not overly rely on the system; if your vehicle is in danger of collision, take all necessary collision-evading actions, such as de- pressing the brake pedal strongly regardless of whether the system is activated or not.” (Mitsubishi ASX, pg. (6)47)
	“WARNING: On rare occasions, detection issues can occur due to the road infrastructures, for example bridges, tunnels and safety barriers. In these cases, the system may brake late or unexpectedly. At all times, you are responsible for controlling your vehicle, supervising the system and intervening, if required.” (Ford Ranger, pg. 186)
*Theme 5*. *User manual informs users of how the ADAS will change their behaviour (behavioural adaptation)*	“The Smart Cruise Control System is not a substitute for safe driving practices, but a convenience function only. It is the responsibility of the driver to always check the speed and distance to the vehicle ahead.” (Hyundai i30, pg. (5)138)
	“**Do not rely completely on the MRCC:**
	The MRCC system has detection limitations depending on the type of vehicle ahead and its conditions, the weather conditions, and the road conditions. Additionally, the system may be unable to decelerate sufficiently to avoid hitting the vehicle ahead if the vehicle ahead applies the brakes suddenly or another vehicle cuts into the driving lane, which could result in an accident. Always drive carefully and verify the surrounding conditions and depress the brake pedal or accelerator pedal while keeping a safer distance from vehicles ahead or on-coming vehicles.” (Mazda 3, pg. (4)138)
	*“*A driver is responsible for driving safely. The FCM is the system to mitigate collision-caused damages or to avoid collisions as much as possible. The system is not intended to compensate for driver’s loss of attention to the front during driving due to distraction or carelessness or supplement a drop in visibility due to the rain and fog.
	It is never a substitute for your safe and careful driving. Always be ready to apply the brakes manually.” (Mitsubishi ASX, pg. (6)46)
	“Driving safely is the sole responsibility of the driver. Do not rely solely on the system, and drive safely by always paying careful attention to your surroundings.” (Toyota Landcruiser, pg. 259)
*Theme 6*. *User manual warns users about confusion when using the system*	“During normal cruise control operation, when the SET switch is activated or reactivated after applying the brakes, the cruise control will activate after approximately 3 seconds. This delay is normal.” (Hyundai i30, pg. (5)133)
	“**Note**: When adaptive cruise control is active, the speedometer may vary slightly from the set speed displayed in the information display.” (Ford Ranger, pg. 183)
	“The sound of the automatic brakes operating may be heard, however, it does not indicate a problem.” (Mazda CX5, pg. (4)168)
*Theme 7*. *User manual instructs users how to respond to a malfunction*	“If the Smart Cruise Control is cancelled by other than the reasons mentioned, we recommend that the system be checked by a HYUNDAI authorised repairer.” (Hyundai i30, pg. (5)143)
	“If there is a malfunction in the system, a warning will appear on the information screen of the multi-information display depending on the situation.
	When the FCM system determines that its performance has been degraded, the FCM will become inoperative.” (Mitsubishi ASX, pg. (6)52)
	“**If “Cruise Control Malfunction Visit Your Dealer” is shown on the multi- information display;**
	Press the “ON-OFF” button once to deactivate the system, and then press the button again to reactivate the system.
	If the cruise control speed cannot be set or if the cruise control cancels immediately after being activated, there may be a malfunction in the cruise control system. Have the vehicle inspected by your Toyota dealer.” (Toyota Landcruiser, pg. 272)

**Table 3:** Summary of qualitative analysis of ADAS by vehicle and theme.

		Informing differences	Familiarisation	Operational limits	Potential ADAS errors	Behaviour adaptation warnings	Confusion warnings	Malfunction warning
Hyundai i30	Cruise control	No	Yes	Yes	Yes	Yes	Yes	Yes
	Blind Spot Monitoring	No	No	Yes	Yes	Yes	No	Yes
Ford Ranger	Cruise control	No	No	Yes	Yes	Yes	Yes	No
	Blind Spot Monitoring	No	No	Yes	Yes	Yes	No	Yes
Mazda CX5	Cruise control	No	No	Yes	Yes	Yes	Yes	Yes
	Blind Spot Monitoring	Yes	No	Yes	Yes	Yes	No	Yes
Mazda 3	Cruise control	No	No	Yes	Yes	Yes	Yes	No
	Blind Spot Monitoring	Yes	No	Yes	Yes	Yes	No	No
Mitsubishi ASX	Cruise control	Yes	No	Yes	Yes	Yes	Yes	Yes
	Blind Spot Monitoring	No	Yes	No	Yes	Yes	No	Yes
Mitsubishi Outlander	Cruise control	No	No	Yes	Yes	Yes	Yes	Yes
	Blind Spot Monitoring	No	Yes	Yes	Yes	Yes	Yes	Yes
Toyota Hilux	Cruise control	No	No	Yes	Yes	No	Yes	No
	Blind Spot Monitoring	N/A	N/A	N/A	N/A	N/A	N/A	N/A
Toyota Landcruiser	Cruise control	No	No	Yes	Yes	Yes	Yes	Yes
	Blind Spot Monitoring	No	No	Yes	Yes	Yes	No	Yes
Toyota Corolla	Cruise control	No	No	Yes	Yes	Yes	Yes	Yes
	Blind Spot Monitoring	N/A	N/A	N/A	N/A	N/A	N/A	N/A
Total	Cruise control	11.11%	11.11%	100.00%	100.00%	88.89%	100.00%	66.67%
	Blind Spot monitoring	28.57%	28.57%	85.71%	100.00%	100.00%	14.29%	85.71%

### 3.2. Quantitative analysis

In this section the number of diversions, presence of infographics and readability were analysed. Regarding diversions (see Table 4), the average number of section diversions within all cruise control sections and all blind spot monitoring sections was found to be 2.44 and 2.86 respectively. Regarding infographics, the illustration numbers ranged from 2 illustrations in the Mazda 3 cruise control section to 21 in the Hyundai i30 blind spot monitoring section. The Ford Ranger was the vehicle with more warning labels (18) on the cruise control section. Overall, the manuals had very similar number of illustrations, icons, and warning labels between cruise control and blind spot monitoring (See Table 5).

**Table 4:** Quantitative analysis of section diversion.

		Total Pages (manual)	Total Pages (section)	Section page numbers	Section Diversion
Hyundai i30	CC	684	6	(5)132 –(5)137	0
	BSM		12	(5)70 –(5)81	1
Ford Ranger	CC	511	8	181–188	1
	BSM		7	195–201	6
Mazda CX5	CC	820	7	(4)-235 - (4)241	5
	BSM		6	(4)126 –(4)131	2
Mazda 3	CC	732	5	(4)195-(4)199	0
	BSM		6	(4)118 –(4)123	3
Mitsubishi ASX	CC	490	6	(6)41 –(6)46	1
	BSM		5	(6)55 –(6)59	3
Mitsubishi Outlander	CC	602	5	(6)70 –(6)74	1
	BSM		7	(6)107 –(6)113	1
Toyota Hilux	CC	676	5	314–318	1
	BSM		N/A	N/A	N/A
Toyota Landcruiser	CC	608	3	270–272	6
	BSM		14	344–357	4
Toyota Corolla	CC	608	12	235–246	7
	BSM		N/A	N/A	N/A
**M (SD)**	CC	636.78 (104.07)	6.33 (2.55)		2.44 (2.74)
	BSM		8.14 (3.44)		2.86 (1.77)

Note: CC = Cruise Control, BSM = Blind Spot Monitoring, M = Mean, SD = Standard Deviation

**Table 5:** Quantitative analysis of infographics.

Vehicle	ADAS	Illustrations	Icons	Warning labels
Hyundai i30	CC	10	0	1
	BSM	21	0	2
Ford Ranger	CC	7	21	18
	BSM	9	0	4
Mazda CX5	CC	4	1	2
	BSM	8	1	1
Mazda 3	CC	2	2	2
	BSM	8	1	1
Mitsubishi ASX	CC	11	0	4
	BSM	5	4	6
Mitsubishi Outlander	CC	11	2	3
	BSM	5	4	5
Toyota Hilux	CC	5	0	1
	BSM	N/A	N/A	N/A
Toyota Landcruiser	CC	5	0	1
	BSM	14	6	3
Toyota Corolla	CC	19	0	2
	BSM	N/A	N/A	N/A
**M (SD)**	CC	8.22 (5.17)	2.89 (6.85)	3.78 (5.43)
	BSM	10.00 (5.72)	2.29 (2.36)	3.14 (1.95)

Note: CC = Cruise Control, BSM = Blind Spot Monitoring, M = Mean, SD = Standard Deviation

The readability of each manual was calculated using an online readability software that generated a score on the Gunning Fog index of readability (Table 6). The readability analysis of all vehicle manuals presented a Gunning Fog index of 11.46, which indicates that 11.46 years of formal education are required to read and comprehend the vehicle manual on the first read-through. The overall difference in readability between the cruise control sections and the blind spot monitoring sections was minimal, with each scoring a Gunning Fog index of 11.41 and 11.52 respectively. The highest Gunning Fog index score was found the be the end part of the cruise control section of the Hyundai i30 manual. In contrast, the lowest Gunning Fog index was found to be the middle section of the blind spot monitoring section of the Mitsubishi Outlander. The Toyota Hilux and the Toyota Corolla are not equipped with blind spot monitoring systems; thus, no data was analyses for the blind spot monitoring system of those two vehicles.

**Table 6:** Readability analysis of vehicle manuals.

	Cruise Control				Blind Spot Monitoring			
Vehicle	Beginning	Middle	End	Mean (SD)	Beginning	Middle	End	Mean (SD)
Hyundai i30	11.81	12.75	23.05	15.87 (6.24)	10.20	12.34	10.01	10.9 (1.29)
Ford Ranger	7.10	11.37	9.18	9.22 (2.14)	12.62	11.89	9.98	11.5 (1.36)
Mazda CX5	19.78	8.57	10.25	12.87 (6.05)	12.11	19.19	14.20	15.2 (3.64)
Mazda 3	17.59	11.39	9.44	12.81 (4.26)	11.79	11.51	12.99	12.1 (0.79)
Mitsubishi ASX	8.79	8.92	12.58	10.10 (2.15)	11.59	11.58	9.78	11.0 (1.04)
Mitsubishi Outlander	9.02	11.58	12.34	10.98 (1.74)	13.91	7.42	9.90	10.4 (3.27)
Toyota Hilux	9.68	9.61	10.88	10.06 (0.71)	-	-	-	-
Toyota Landcruiser	12.31	8.34	9.84	10.16 (2.00)	10.20	10.15	8.52	9.6 (0.96)
Toyota Corolla	12.87	8.29	10.69	10.62 (2.29)	-	-	-	-
**M (SD)**	12.11 (4.20)	10.09 (1.69)	12.03 (4.30)	11.41 (2.08)	11.77 (1.32)	12.01 (3.57)	10.77 (2.03)	11.52 (1.80)
**Overall M (SD)**	11.46 (0.71)							

Note: M = Mean, SD = Standard Deviation.

## 4. Discussion

The present investigation reports on a content analysis of the ADAS-related information present in the user manuals of the highest selling cars in Australia in 2018. Previous research has demonstrated the potential safety gains from these new systems [18, 19]. However, the adoption of ADAS brings new challenges for driver education and training. Specifically, there are concerns about misconceptions or lack of awareness of ADAS functionalities among drivers [20]. For example, Harms et al. [8] found that the majority (65%-83%) of Dutch drivers of vehicles with ADAS systems such as adaptive cruise control, lane departure warning, emergency brake, and distance alert systems were mostly unaware of owning ADAS. Debate continues about the best strategies to educate drivers about these systems. Currently, most of the existing education relies on drivers reading the user manuals and complementing this with other sources of information such as online forums. Additionally, previous research shows that the majority of drivers use trial-and-error and discuss the systems with friends or family as opposed to reading the user manual [8, 21]. It has been suggested that this occurs because user manuals can be difficult to understand, too long, and contain insufficient information [9]. This indicates a need to understand the content and characteristics of the user manuals.

Our results confirm that the content of some of the manuals includes a wide range of important themes for the safe use of ADAS. Some of the themes, covered by most of the vehicle manuals (> 80%), are critical to warrant safe use of the ADAS, i.e., operational limits, potential ADAS errors, and behaviour adaptation warnings. For example, previous research has shown that drivers can misuse ADAS systems and reduce protective behaviours as a consequence of the ADAS [9, 21, 22], which could result in safety risks given that the ADAS have important limitations as highlighted in the user manuals (See Tables 1 and 2). However, it is curious that a higher proportion of user manuals did not include information on the differences between driving with and without the ADAS, the need for familiarisation, warnings about potential confusion because of the ADAS, and malfunctions of the ADAS. This result was very surprising since we would expect these themes to be relevant for all vehicles and types of ADAS. Thus, there is no reason for not having this information consistent in all manuals for all systems. The inconsistent information presented in the user manuals suggests that drivers are receiving irregular education about ADAS. This could be symptomatic of a weak standard for baseline knowledge on the ADAS. Moving forward, there is a need to understand what drivers require to be sufficiently educated to use their ADAS safely. Both regulators and industry, ideally with the participation of broader transport stakeholders, should clarify this gap. Research has shown the need for more leadership from government regarding the education of in-vehicle technologies [20].

The findings from the present research show that user manuals have safety-critical information about using ADAS, which is consistent with earlier research [23]. Whilst manuals have safety-critical information, the recommendation of reading the manual before operating the vehicle’s equipped systems has not been widely acknowledged by road authorities. For example, the only indication from the Queensland Government [24] for users to read the operations manual for their vehicle is found on a page flagged for vehicle maintenance and upkeep. However, the low readership of manuals is not exclusive to car drivers. For example, Blackler, Gomez, Popovic and Thompson [25] found that people do not read the manuals provided with many of their home appliances. More importantly, the authors found that this led to a significant number of people being unaware of the functionalities included within their own devices. The previous study, while not concerning ADAS, demonstrates the limitations of relying exclusively on an individual to read and understand their user manual as the only means of developing an effective mental model. There is a need to develop complementary strategies dedicated to upskilling drivers in the use of ADAS in addition to or even apart from the user manual.

Manuals also include several human factors issues regarding the number of times a reader must divert around the manual to find information and the wide range of infographics that must be interpreted and understood. Diversions in a user manual can create challenges effecting the text flow as well as distractions from the ADAS. In this study, we found that in some vehicles the number of diversions was high, which decreases readability. If the automobile industry expects that education about their systems be solely or primarily based on user manuals, they should try to improve them. Similarly, we identified a wide range of imagery used in manuals which can also significantly erode a reader’s efforts to understand and put into practice a manual’s content. Relevant to this, Thompson et al. [3] found ambiguity around ADAS alert symbols and their meaning. This is a significant issue when considering that many drivers have access to ADAS through renting vehicles or having multiple cars in a household (extremely common in car-centric jurisdictions such as Australia). The lack of familiarity with the imagery can result in confusion or erroneous mental models of the ADAS. Previous research has demonstrated the need for accurate mental models of the ADAS to guarantee safe driving [23].

A key finding of this study is that to be able to both read and comprehend the manuals a high number of years of education is required. Some of the vehicles and ADAS require several years of education above the recommended for a universal audience (>8). This creates limitations in the understanding of the manuals particularly among young drivers, people with disability, people with limited English proficiency, or those who have not completed the assumed level of education required. Existing literature has examined the difficulty faced by users to read and understand manuals based on the language and grammar used to relay important information. Smart et al. [26] found that users have difficulty understanding user manuals due to confusing and inconsistent terminology, as well as the manuals making incorrect assumptions about what the reader knew. Wallace and Keenum [15] examined different quick reference guides and user manuals for 22 other brands of home blood pressure monitors. It was found that many of the manuals did not meet the readability standard, wherein the manual required a reading grade of eight to ten years of formal education to be read. The recommendation for readability for such user manuals was between six to eight years of formal education. This further reinforces the idea that manuals should not be the only strategy to educate and upskill individuals to use technology safely.

### 4.1. Limitations

The present manuscript has a number of limitations. Firstly, car models considered in this study were top sellers in Australia, which may not represent the features available in other jurisdictions. Next, we only analysed the content of user manuals of vehicles, but the users can also obtain information from the internet, or from friends and family. That information can have varying levels of accuracy, and it is unclear if it can correctly complement, or indeed undermine, the user manuals. Similarly, only nine of the ten vehicles listed were able to be examined, due to the unavailability of the Mitsubishi Triton manual online, and the lack of an 11^th^ vehicle from the list used to select the vehicles. Also, in the quantitative analysis, the Gunning Fog Index is based on a formula which determines the complexity of a word based on the number of syllables contained within the said word. As a result, commonly used words that contain three syllables are valued as ‘more complex’ than a word with two syllables that is less well known or has a definition that is more difficult to understand. This index may not precisely represent the complexity of the text. With this in mind, it is important to recognise that this investigation did not analyse perceptions of users directly. By obtaining direct information from human subjects about their experience using the manuals, we may gain additional understanding of readability and usability of the manuals. In addition, studies using human subjects can help to understand the actual impact that interactions with manuals have on road user behaviour. Also, the present study only analysed the technology component of user manuals. This means that we cannot conclude whether the presence of this technology is the reason for why the manuals are complex to read or not. Future research should seek to compare vehicle manuals with and without ADAS to understand the impact that the technology has on readability of the manuals. Generally, additional studies are needed to broaden our understanding of the gaps and best practice of ADAS education policy.

## 5. Conclusions

To conclude, the information presented in the user manuals of vehicles with ADAS is safety-critical for driving. However, the large variability in the content of the manuals highlights the need for benchmarks of the minimum education needed to drive with ADAS safely. Another critical issue is that the manuals present several potential usability issues such as large numbers of text diversions and infographics. It was also found that these manuals are generally written to be understood by a highly educated audience. These issues likely decrease the usability of the manuals, which could potentially contribute to low readership. This investigation shows that there is a lack of standardisation of ADAS user manuals (in both content and delivery of information) which requires regulatory oversight.

## Supporting information

S1 Appendix (DOCX)
