# Supplementary material for: Is a flick-through enough? A content analysis of Advanced Driver Assistance Systems (ADAS) user manuals
Source: PLoS One. 2021 Jun 17;16(6):e0252688. doi: 10.1371/journal.pone.0252688 (PMC8211250; doi:10.1371/journal.pone.0252688)
Supplement: S1 Appendix — (DOCX) [file pone.0252688.s001.docx]

**S1 Appendix**

Source files for the ADAS manuals used for this study can be found as below.

| **Vehicle make/model** | **Source** |
| --- | --- |
| Hyundai i30 | <https://www.hyundai.ee/ru/wp-content/uploads/2017/04/i30-manuals-ENG.pdf> |
| Ford Ranger | [https://www.fordservicecontent.com/Ford_Content/vdirsnet/OwnerManual/Home/Index?](https://www.fordservicecontent.com/Ford_Content/vdirsnet/OwnerManual/Home/Index?Variantid=6124&languageCode=EN&countryCode=USA&marketCode=US&bookcode=O39988&VIN=&userMarket=AUS&div=f)  [Variantid=6124&languageCode=EN&countryCode=USA&marketCode=US&bookcode=](https://www.fordservicecontent.com/Ford_Content/vdirsnet/OwnerManual/Home/Index?Variantid=6124&languageCode=EN&countryCode=USA&marketCode=US&bookcode=O39988&VIN=&userMarket=AUS&div=f)  [O39988&VIN=&userMarket=AUS&div=f](https://www.fordservicecontent.com/Ford_Content/vdirsnet/OwnerManual/Home/Index?Variantid=6124&languageCode=EN&countryCode=USA&marketCode=US&bookcode=O39988&VIN=&userMarket=AUS&div=f)  [https://www.fordservicecontent.com/Ford_Content/Catalog/owner_information/](https://www.fordservicecontent.com/Ford_Content/Catalog/owner_information/JB3B19A285ZAA-2018-P375-Ranger-Raptor-TKD-SUPP-ENG-AUSandNZL-2019.pdf)  [JB3B19A285ZAA-2018-P375-Ranger-Raptor-TKD-SUPP-ENG-AUSandNZL-2019.pdf](https://www.fordservicecontent.com/Ford_Content/Catalog/owner_information/JB3B19A285ZAA-2018-P375-Ranger-Raptor-TKD-SUPP-ENG-AUSandNZL-2019.pdf) |
| Mazda CX5 | <https://owners-manual.mazda.com/gen/en/cx-5/cx-5_8ft1ee17b/howto.html> |
| Mazda 3 | <https://owners-manual.mazda.com/gen/en/mazda3/mazda3_8fj4ee16e/howto.html> |
| Mitsubishi ASX | <https://owners.mitsubishi-motors.co.uk/media/pdfs/owners-manuals/OGAE18E1.pdf> |
| Mitsubishi Outlander | <https://owners.mitsubishi-motors.co.uk/media/pdfs/owners-manuals/OGFE18E1.pdf> |
| Toyota Hilux | <https://toyotamanuals.com.au/document/landing_page/hilux-owners-manual-dec-17-jun-18> |
| Toyota Landcruiser | <https://toyotamanuals.com.au/document/landing_page/landcruiser-200-owners-manual-aug-17-aug-18> |
| Toyota Corolla | <https://toyotamanuals.com.au/document/landing_page/corolla-owners-manual-nov-16-jun-18> |
